# Compressive Deformation Behavior of Closed-Cell Micro-Pore Magnesium Composite Foam

**DOI:** 10.3390/ma11050731

**Published:** 2018-05-04

**Authors:** Jing Wang, Nannan Wang, Xin Liu, Jian Ding, Xingchuan Xia, Xueguang Chen, Weimin Zhao

**Affiliations:** 1 School of Material Science and Engineering, Hebei University of Technology, Tianjin 300130, China; w1225377693@163.com (J.W.); w13820962170@163.com (N.W.); liulz3.1415926@vip.163.com (X.L.) djian0122@126.com (J.D.); cxg@hebut.edu.cn (X.C.); 2 School of Material Science and Engineering, Tianjin University, Tianjin 300354, China

**Keywords:** magnesium composite foam, ceramic microspheres, compressive behavior

## Abstract

The closed-cell micro-pore magnesium composite foam with hollow ceramic microspheres (CMs) was fabricated by a modified melt foaming method. The effect of CMs on the compressive deformation behavior of CM-containing magnesium composite foam was investigated. Optical microscopy and scanning electron microscopy were used for observation of the microstructure. Finite element modeling of the magnesium composite foam was established to predict localized stress, fracture of CMs, and the compressive deformation behavior of the foam. The results showed that CMs and pores directly affected the compressive deformation behavior of the magnesium composite foam by sharing a part of load applied on the foam. Meanwhile, the presence of Mg_2_Si phase influenced the mechanical properties of the foam by acting as the crack source during the compression process.

## 1. Introduction

Metal matrix syntactic foams (MMSF) are a novel class of materials which possess excellent mechanical properties compared to traditional metal foams, e.g., high strength-to-weight ratio, high specific stiffness, excellent thermal insulation, and low thermal expansion coefficient [1,2,3,4]. Therefore, metal matrix syntactic foams are candidates for weight-sensitive applications in transport, ship engineering, aerospace, and construction fields [5,6,7]. Up to now, preparation methods, foaming mechanisms, and mechanical properties of MMSF have been widely explored in the available literature. Most of the investigations used alumina [8], SiC particles [9], fly ash [10,11], glass cenospheres, or other ceramic particles [12,13] as the fillers. Closed-cell magnesium composite foam is a kind of newly-developed metal foam, which contains a certain quantity of hollow ceramic microspheres (CMs, a by-product of coal combustion in thermal power plants). In particular, magnesium composite foam has been drawing much attention in construction, automotive, and aerospace applications from the viewpoint of environmental preservation due to its unique combination of thermal, physical, acoustic, mechanical, and electrical properties [14,15,16]. 

To date, methods for the preparation of magnesium composite/alloy foams have been developed, such as the powder metallurgy method, the infiltration casting method, and the melt foaming method. The melt foaming method is widely used due to its unique advantages [17,18]. To date, the average pore size of traditional closed-cell magnesium alloy foam prepared by the melt foaming method is mainly distributed in the range of 1.5–2.5 mm, and generally a single thickening agent (mainly Ca, Al, or SiC) was used [18,19]. Magnesium alloy foams with an average pore size smaller than 1 mm have seldom been reported. However, it has been confirmed that smaller pore size is beneficial to its compression process stability and strength [16]. Meanwhile, smaller cell size contributes to the acoustic performance improvement of metal foams [20]. 

Among all of the mechanical properties, compressive behavior is an important indicator to evaluate the basic mechanical property of cellular materials. Therefore, compression characteristics of metal foams have been studied in numerous aspects. Orbulov investigated the effect of hollow sphere size and the aspect ratio of specimens on the failure mechanisms and mechanical properties of aluminum matrix syntactic foams [21]. Brila et al. studied the compressive deformation behavior of aluminum-cenosphere hybrid foam with varying amounts of cenospheres [22]. Myers et al. prepared aluminum alloy matrix syntactic foam by inert gas pressure infiltration and found that their compressive strength showed limited sensitivity to strain rate [23]. Santa Maria et al. discussed the effect of hollow sphere dimensions and volume fractions on the compressive property of aluminum matrix syntactic foam and the results showed that the peak strength, plateau strength, and toughness of the foam increased with decreasing hollow sphere wall thickness to diameter (t/D) ratio [24]. As described above, most of investigations focused on the compressive characteristics of aluminum matrix syntactic foams. Few researchers have paid attention to the compressive deformation behavior of magnesium composite foams. Daoud et al. and Huang et al. reported the microstructure and compressive property of magnesium/fly-ash cenosphere composite foam and concluded the relationship between the compressive behavior and the content of fly ash cenospheres [25,26]. In our previous work, a modified melt foaming method was applied to successfully prepare closed-cell magnesium composite foam [13]. Meanwhile, the compressive property was studied and the results showed that CMs could change the fracture mode of magnesium composite foam. However, the fracture mechanism of magnesium composite foam under compression conditions was not clear and further research is needed, which is beneficial to its application as a structural material. 

The aim of this paper is to prepare closed-cell micro-pore magnesium composite foam with CMs by the modified metal foam method. Meanwhile, the effect of CMs, pores, and the Mg_2_Si phase on the compressive deformation behavior and associated failure mechanism of the foams were investigated and discussed

## 2. Experimental Details

### 2.1. Specimens Preparation

The closed-cell magnesium composite foam was fabricated by the modified melt foaming method. Commercial AZ31 magnesium alloy (the composition as shown in Table 1), hollow ceramic microspheres (CMs, thickening agent, the composition and parameter as shown in Table 2), Ca particles (with diameters of 1–2.5 mm, thickening agent), and CaCO_3_ (analytically pure, foaming agent) were used as raw materials. Detailed preparation process were as follows: (1) cutting a certain quantity of a commercial AZ31 magnesium ingot into sheets with a thickness of 10–15 mm by electro-discharging machining; (2) stacking the sheets and CMs layer by layer in a low-carbon steel crucible; (3) melting the layered materials in the low-carbon steel crucible at 953 ± 5 K and then holding for 20 min; (4) adding 0.5 wt % Ca particles to the melt with an impeller stirring speed of 500 r/min for 8 min; (5) adding 1.5 wt % CaCO_3_ powders to the melt accompanied with the impeller stirring speed of 1200 r/min for 30 s; and (6) holding the melt for 2 min and then cooling the foam in air. For comparison, AZ31 magnesium foam (without CMs) and AZ31 magnesium composite material (without a thickening agent and CaCO_3_ powders) were prepared. For the AZ31 magnesium foam (without CMs), the processing parameters were coincident, except for the content of Ca particles, the content of which increased to 2 wt %. For the AZ31 magnesium composite material (without a thickening agent and CaCO_3_ powders) the processing parameters were also coincident. SF_6_ and CO_2_ mixture gas (with volume ratio of 1:100) was used to protect the melt from being oxidized.

### 2.2. Compression Test

Quasi-static compression tests were performed on a SUN Electron Universal Testing Machine (Shenzhen SUNS Technology Stock CO., LTD, Shenzhen, China) fitted with a 300 kN load cell at room temperature. Specimens for compression tests were cut into the size of 25 × 25 × 25 mm (length × width × thickness) by electro-discharging machining. The tests were controlled by displacement with a constant speed of 1.5 mm/min (with initial strain rate of 0.001/s). Vaseline was used as the lubricant to minimize the friction between the specimen and plates. Three specimens were tested for each material. A set of interrupted compressive tests (with strains of 0.05 and 0.2, respectively) were performed to observe the deformation behavior of the CMs and matrix.

### 2.3. Microstructure Observation

Typical metallographic preparation processes were used for microstructure observation. The microstructure of the magnesium composite foam/material and the magnesium alloy foam were characterized by optical microscope (OM, Zeiss LSM 800, Carl Zeiss, Oberkochen, Germany) and scanning electron microscope (SEM, Nova Nano 450, FEI, Hillsboro, OR, USA) equipped with an energy dispersive X-ray spectrometer (EDS). 

### 2.4. Finite Element Modeling

3D finite element model of a cubic representative elementary volume (0.2 × 0.2 × 0.2 mm) of the magnesium composite foam/material was established, an implicit solver in ABAQUS 6.14-3 was used to predict the localized stress field and deformation behavior of CMs, pores, and the magnesium matrix. Detailed parameters were as follows: the Young’s modulus of E_CM_ = 72 GPa and Poisson ratio of ν_CM_ = 0.18 for the CMs, and E_Mg_ = 42 GPa and ν_Mg_ = 0.35 for the magnesium matrix [27,28].

## 3. Results and Discussion

### 3.1. Morphology Observation

**Figure 1:**
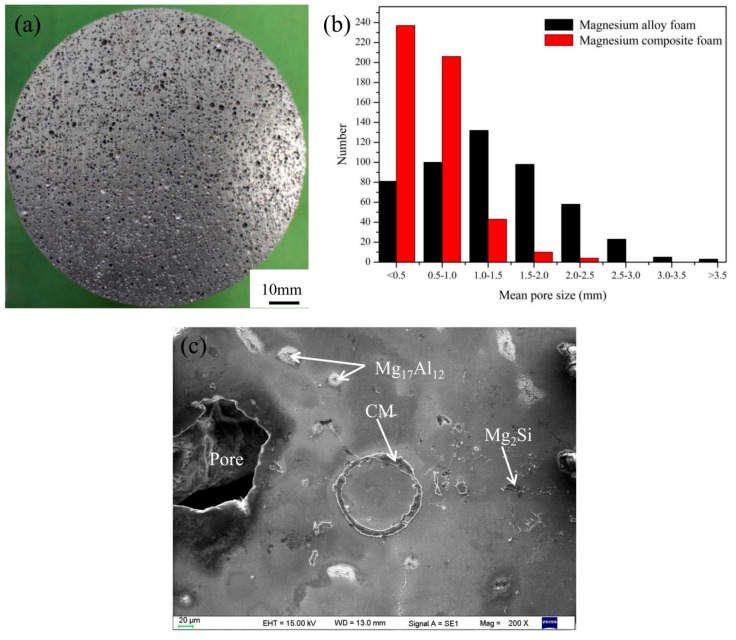
Typical macrostructure (**a**), pore sizes distribution (**b**), and the microstructure (**c**) of magnesium composite foam.

Figure 1 shows the typical macrostructure, pore size distribution, and microstructure of the magnesium composite foam. It can be seen that the spherical and separated pores distribute homogeneously in the magnesium composite foam (Figure 1a). The pore size mainly distributes in the range of 0.1–2.5 mm and only about ten percent of pores are larger than 1 mm (Figure 1b), the pores with diameters of 3–4 mm disappear. It has been known that the pores of the magnesium alloy foam mainly distribute in the range of 0.3–4.0 mm and about 60% of pores are larger than 1 mm (Figure 1b) [29], indicating that composite thickening with CMs and Ca can obviously reduce the pore size and improve the homogeneity of pores. In addition, it can be clearly seen that CMs with the original morphology embed in the cell walls. Meanwhile, based on our previous research, Mg_2_Si and Mg_17_Al_12_ distribute in the cell walls [30].

### 3.2. Compression Characteristics 

**Figure 2:**
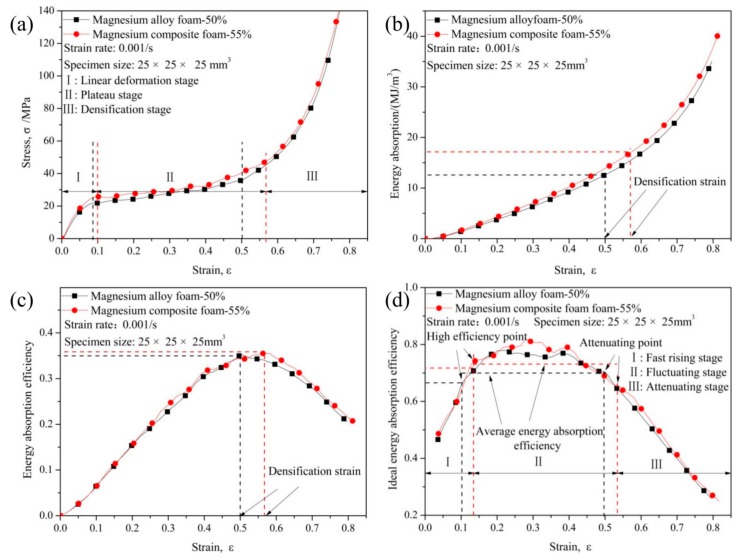
Compressive characteristics of magnesium alloy foam and magnesium composite foam: stress-strain curves (**a**), energy absorption capacity (**b**), energy absorption efficiency (**c**), and ideal energy absorption efficiency (**d**).

Figure 2 shows the compressive characteristics of magnesium composite foam and magnesium alloy foam. Generally speaking, the stress-strain curve of metal foam (magnesium composite foam is used as a demonstration) mainly consists of a linear deformation stage (I) where the stress increases almost linearly with increasing strain, a plateau deformation stage (II) with stress nearly constant along with increasing strain, and a densification stage (III) where the stress increase steeply with increasing strain. As is well established, yield stress is important for estimating the mechanical properties of metallic foams. In this paper, the first peak stress on stress-strain curve is defined as the yield strength. Yield strength of the magnesium composite foam is about 25.75 MPa, which is much higher than that of the magnesium alloy foam (21.18 MPa). Typically, the yield strength of metal foam decreases with increasing porosity [6]. However, in this paper, the porosity of magnesium composite foam is about 55%, which is higher than the magnesium alloy foam (with porosity of about 50%), meaning that CMs could significantly improve the yield strength of the magnesium alloy foam. 

Energy absorption capacity (Equation (1)), energy absorption efficiency (Equation (2)), and ideal energy absorption efficiency (Equation (3)) are important aspects to evaluate the mechanical properties of metal foams [31,32,33,34]:

(1) $$W=\int_{0}^{\varepsilon }\sigma d\varepsilon ,$$

(2) $$E=\frac{W}{\sigma },$$

(3) $$I=\frac{W}{\sigma \varepsilon },$$

where *W* is the energy absorption capacity, *E* is the energy absorption efficiency, *I* is the ideal energy absorption efficiency, and *σ* is the stress where the strain is *ε*. Plateau stress, defined as the average stress within the strain range of 0.1 to 0.5, is taken into account to evaluate the energy absorption capacity of metal foam. As shown in Table 3, plateau stress increases from 27.93 MPa to 30.6 MPa after adding CMs into the magnesium alloy foam. In addition, it can be clearly seen from Figure 2b that energy absorption capacity increases with strain increasing. Furthermore, energy absorption capacity is increasing almost the same for the two foams at the initial stage. However, the differences are distinct in the middle stage: the energy absorption capacity of magnesium composite foam and magnesium alloy foam are about 10.23 and 9.28 MJ/m^3^ when the strain is 0.4, respectively. It should be noted that in the middle stage the energy absorption capacity of the magnesium composite foam is higher than the magnesium alloy foam, which could be attributed to the magnesium composite foam possessing a higher yield strength and plateau stress (as shown in Table 3).

As shown in Figure 2c, energy absorption efficiency of the two kinds of foams increases firstly, and then decreases with increasing strain. Generally speaking, the strain corresponding to the highest energy absorption efficiency is defined as the densification strain [35]. The highest energy absorption efficiency of magnesium composite foam is 0.355 with a densification strain of 0.57, while for magnesium alloy foam, the highest energy absorption efficiency is 0.35 with the densification strain of 0.5, meaning that the energy absorption efficiency decreases after adding CMs into the magnesium alloy foam. Furthermore, the energy absorbed until the densification strain is defined as the total energy absorption capacity. As shown in Figure 2b,c, the total energy absorption capacity of the magnesium alloy foam is 12.61 MJ/m^3^ with a densification strain of 0.5, while, for the magnesium composite foam the total energy absorption capacity is 16.96 MJ/m^3^.

As shown in Figure 2d, the idealenergy absorption efficiency (I) curves consist of fast rising stage (I) where the idealenergy absorption efficiency increase rapidly to high efficiency point with strain increasing, sustained stage (II) where the idealenergy absorption mainly keeps in high efficiency level and some fluctuations appear along with the strain increasing, and attenuation stage (III) where the idealenergy absorption efficiency decreases with strain increasing. From the view point of sustained stage, it can be found that the average energy absorption efficiency (0.77) of the magnesium composite foam is higher than that of magnesium alloy foam (0.75) (Figure 2d and Table 3). The ideal energy absorption efficiency of magnesium composite foam reach to 0.74 with the strain is about 0.14, while the magnesium alloy foam is around 0.68 with a strain of about 0.1.

### 3.3. Effect of Pore and CMs on the Compressive Deformation Behavior

**Figure 3:**
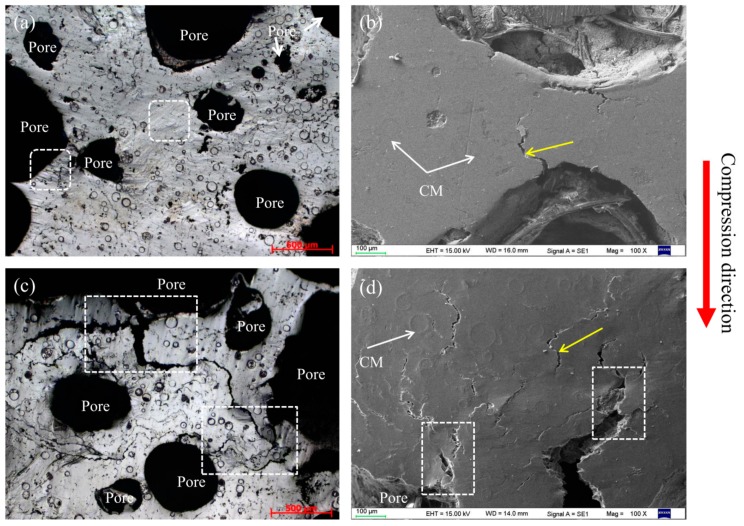
Compressive deformation characteristics of magnesium composite foam with strain of 0.05 (**a**,**b**) and 0.2 (**c**,**d**).

To clarify their compressive deformation behavior, magnesium composite foam undergoes a set of compressive tests at ambient temperature. Figure 3 shows the compressive deformation characteristics of the magnesium composite foam with strain of 0.05 and 0.2. As shown in Figure 3a,b, the cell walls mostly get wrinkles initially and then micro-crack propagation primarily occurs near the wrinkles. The micro-cracks developed to cracks with the compressive stress increasing, resulting in the pores being crushed and compacted (Figure 3c,d). 

**Figure 4:**
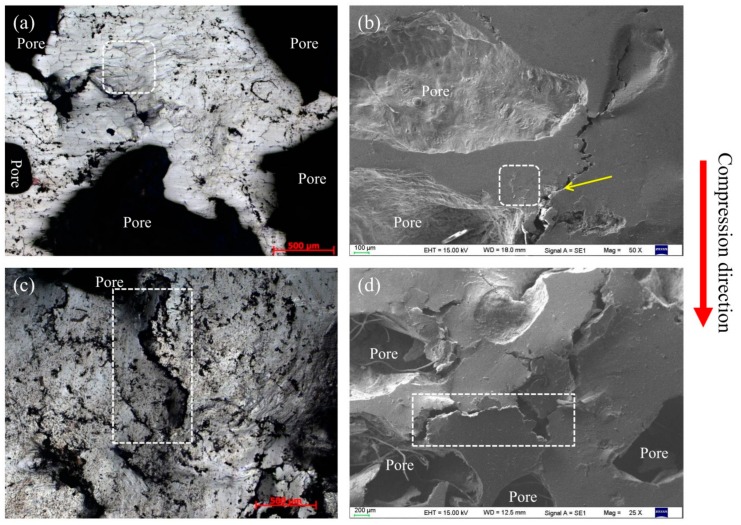
Compressive deformation characteristics of the magnesium alloy foam with strains of 0.05 (**a**,**b**) and 0.2 (**c**,**d**).

**Figure 5:**
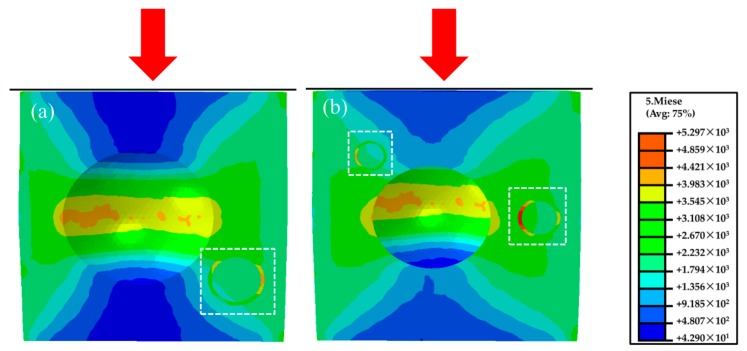
Simulation results of the magnesium composite foam under a strain of *ε* = 0.05, CM located below of pore (**a**), CM located above and the same plane of pore (**b**).

Within the linear deformation stage (*ε* = 0.05), wrinkles and micro-crack appear on the cell wall of the magnesium composite foam (marked by white square and yellow arrow in Figure 3a,b). As can be seen from Figure 3, some sharp corners appear on the pores of the magnesium composite foam, which will lead to the initiation of stress concentration and wrinkles or micro-cracks. With continued increases of the strain (*ε* = 0.2, as shown in Figure 3c,d), wrinkles and micro-cracks evolve into cracks, and the cracks propagate to the cell wall of the magnesium composite foam in the plateau deformation stage. It should be pointed out that the cracks at the cell wall converge on a crack “river” (the white rectangle marked in Figure 3c,d), which finally run through to the adjacent pores, resulting in the pores being crushed and compacted. A similar phenomenon is also observed in magnesium alloy foam (Figure 4). It is interesting that the cracks in the magnesium alloy foam are deeper than the magnesium composite foam under the same condition, indicating that the magnesium alloy foam has poor deformation ability. Moreover, the amount of cracks in the magnesium composite foam is larger and the scale is relatively smaller compared with the magnesium alloy foam. Thus, the magnesium composite foam absorbs more energy per unit area, resulting in the increasing yield stress. Figure 5 shows the numerical simulation results of the overall stress distribution in the magnesium composite foam with a strain of 0.05. It is clear that the effective stress mainly concentrates on CMs and the stress concentration firstly appears on the side of CMs near the pore when the CM and pore are in the same plane (with the square marked in Figure 5), meaning the CM will first fracture under the present conditions. However, stress concentration appears on the side away from the pore when CMs are located above or below of the pore, indicating that the CMs will share part of compressive stress and relieve the compressive stress applied to the pores. All of these are beneficial to delaying the collapse of pores and increasing the compressive strength of the magnesium composite foam. Meanwhile, for individual pores, stress concentration appears near the equator, suggesting that pores will fracture by vertical splitting along the compressive loading axis (as shown in Figure 3). Meanwhile, with the increasing compressive load, vertical splitting will spread to the adjacent pores, resulting in the pores’ collapse.

**Figure 6:**
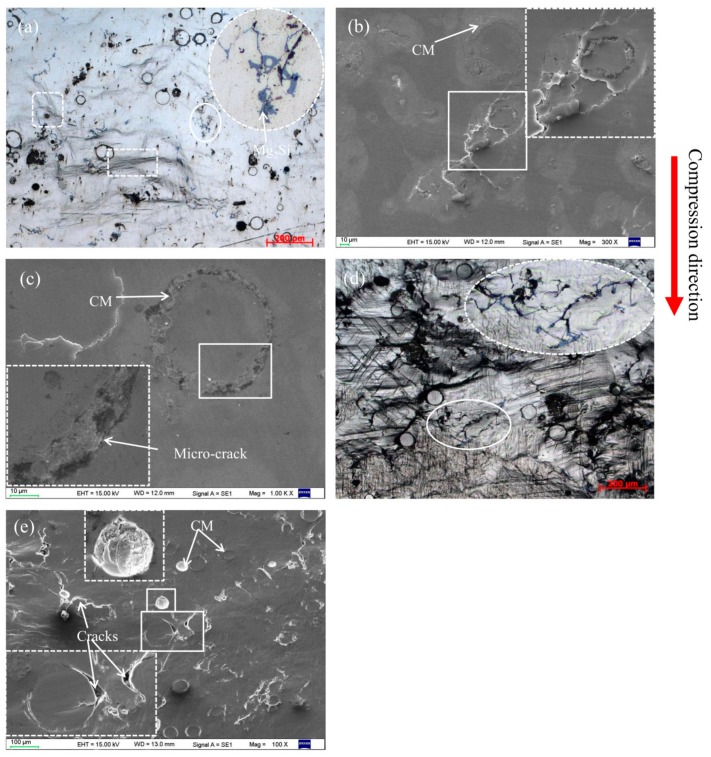
Compressive deformation characteristics of the magnesium composite material with a strain of 0.05 (**a**–**c**) and 0.2 (**d**,**e**).

**Figure 7:**
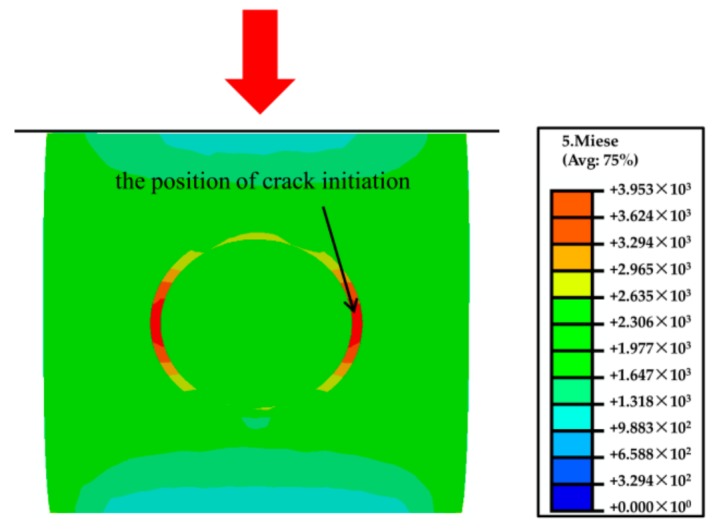
Simulation results of the magnesium composite alloy under a strain of *ε* = 0.05.

In order to better understand the effect of CMs on the fracture behavior of the magnesium composite foam, magnesium composite materials were prepared and a set of compressive tests at ambient temperature were applied. Figure 6 shows the compressive deformation characteristics of the magnesium composite material with a strain of 0.05 and 0.2. Under lower strain (*ε* = 0.05), it can be clearly seen that wrinkles appear in the specimen, like the magnesium composite foam. Meanwhile, micro-cracks primarily occur near the wrinkles and the micro-cracks will bypass the CMs during the propagation process (Figure 6b). Furthermore, micro-cracks were also found along the interface of the CMs and the magnesium matrix (Figure 6c). Under higher strain (*ε* = 0.2), micro-cracks transform into cracks with increasing compressive loading. Micro-cracks along the interface of the CMs and the magnesium matrix will extend with increasing compressive loading, resulting in cleavage along the interfaces (as shown in Figure 6e). This indicates that the interface bonding strength of the CMs and the magnesium matrix is weaker than other areas. More micro-cracks appear in the magnesium composite material at a strain of 0.2, and this is attributed to the existence of CMs, which will increase the amount of cracks and absorb the energy during the compressive deformation process. As a consequence, the growth and coalescence of micro-cracks cause the failure of the magnesium composite material. Figure 7 demonstrates the maximum principal stress field in the magnesium composite material. The localized stress on the CM is considerably higher compared with the other area, which is attributed to the comparatively higher Young´s modulus of the CMs than the magnesium alloy [27,28]. In addition, for individual CMs, stress concentration appears near the annular equatorial area, indicating that CMs will fracture by vertical splitting along the compressive loading axis, which is similar to the magnesium composite foam (Figure 3). A similar phenomenon has been reported on the glass, carbon microballoons, and metallic hollow sphere [36,37,38].

### 3.4. Effect of Mg_2_Si on the Compressive Deformation Behavior

**Figure 8:**
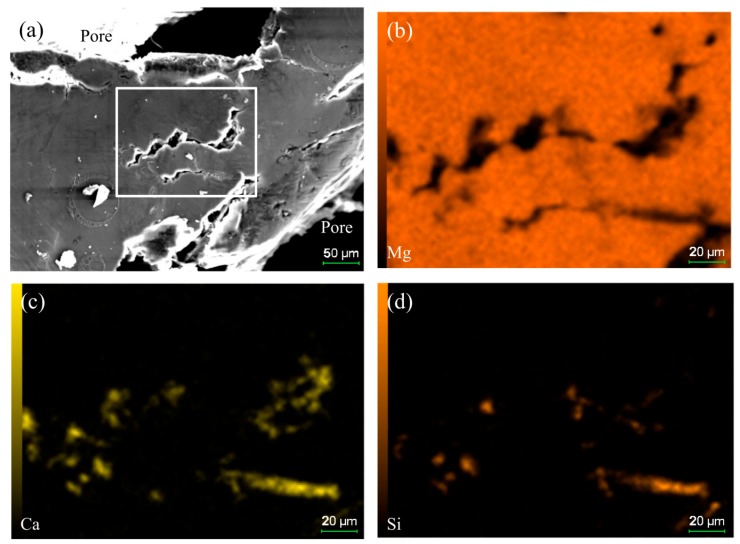
Effect of element distribution on the propagation behavior of compressive cracks, SEM image (**a**) and surface scanning of element Mg (**b**), Ca (**c**) and Si (**d**).

As shown in Figure 1b, the Mg_2_Si phase (due to the reaction of SiO_2_ and magnesium melt) appears on the cell wall of the magnesium composite foam. The Mg_2_Si phase, with irregular block form, is a hard and brittle phase in the magnesium composite foam (Figure 1b), and has a split effect on the magnesium matrix during the compression process [39,40]. Therefore, the Mg_2_Si phase provides a nucleation site of early micro-cracks and acts as a crack source of the magnesium composite foam during the compression process [40,41]. Under lower strain (as shown in Figure 6a), it can be clearly seen that micro-cracks occur on the Mg_2_Si phases while the magnesium matrix round the Mg_2_Si phases has a slight wrinkle. Under higher strain (as shown in Figure 6d), micro-cracks transform into cracks and propagate to the magnesium matrix, resulting in the deboning of the Mg_2_Si phase from the matrix. The wrinkles in the magnesium matrix around the Mg_2_Si phase provide the conditions for the extending of cracks. Figure 8 shows the effect of element distribution on the propagation behavior of compressive cracks. It can be seen that cracks mainly propagate along the area where Ca and Si elements are enriched, suggesting the poisoning effect of the segregation of Ca atoms at the growth front of the Mg_2_Si [42,43]. Meanwhile, the addition of Ca refines the Mg_2_Si phase [44,45] and increases the amount of the Mg_2_Si phase, resulting in the increase of the cracks’ source during the compression process. Thus, compared with the magnesium alloy foam, the amount of cracks increases and the compressive stress applied to the matrix is dispersed, leading to a higher yield strength and energy absorption capacity of the magnesium composite foam.

### 3.5. Failure Mechanism of Magnesium Composite Foam

**Figure 9:**
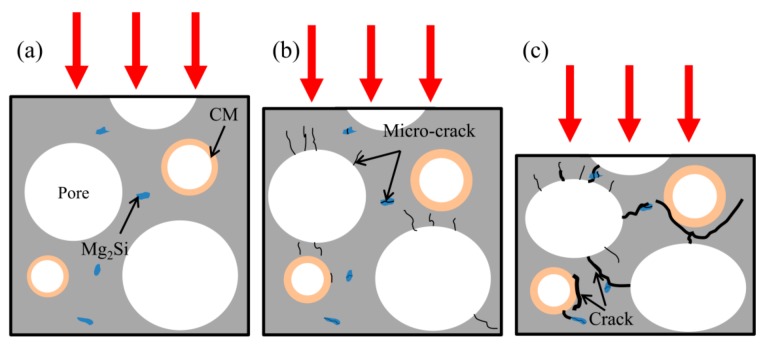
Schematic diagram of crack formation and propagation in the magnesium composite foam with a strain of 0 (**a**), 0.05 (**b**), and 0.2 (**c**).

Figure 9 depicts the crack formation and propagation in the magnesium composite foam. It can be seen that, at the beginning of compressive deformation (Figure 9b), micro-cracks in the matrix are mainly near the pores and the micro-cracks propagate into the cell wall. Meanwhile, some micro-cracks occurred around the Mg_2_Si phase and along the interface of the CM and the magnesium matrix. With the continued increase of the deformation strain into the plateau deformation stage (as shown in Figure 9c), micro-cracks near the pores and the Mg_2_Si phase transform into cracks. Then cracks join neighboring pores and macroscopic cracks form. Meanwhile, micro-cracks along the interface of the CMs and the magnesium matrix will extend with increasing compressive loading, resulting in cleavage of the interfaces. In addition, as CMs and pores carry the majority of the applied load, during the compressive process, stress concentration makes them fracture through vertical splitting along the compressive loading axis and vertical splitting will propagate into the cell wall of the magnesium composite foam to form macroscopic cracks. 

## 4. Conclusions

The compressive deformation behavior of a magnesium composite foam was investigated. The conclusions were drawn as follows: The micro-pore magnesium composite foam (with more than 90% of pore size less than 1 mm) was fabricated by composite thickening technology (using CMs and Ca as the composite thickening agents). CMs improve the yield strength, the plateau stress, and the energy absorption capacity of magnesium alloy foam. Meanwhile, based on the numerical simulation results, CMs and pores carry the majority of the applied load and, during the compression processing stress, mainly concentrates on the equator of CMs and pores. CMs and pores fail by the vertical splitting fracture along the compressive loading axis and vertical splitting propagates into the cell wall of the magnesium composite foam to form macroscopic cracks, resulting in pores crushing and compacting. The Mg_2_Si phase can act as crack sources of magnesium composite foam during the compression process and Ca refines the Mg_2_Si phase, resulting in the improvement of yield strength of the magnesium composite foam. 

## Figures and Tables

**Table 1:** Composition and parameters of CM.

Al_2_O_3_ (wt %)	SiO_2_ (wt %)	Stacking Desity (g/cm^3^)	Size Range (μm)	Wall Thickness (μm)
~10	~90	0.42	40–150	7.5 ± 0.8

**Table 2:** Composition of AZ31B magnesium alloy, wt %.

Al	Zn	Mn	Si	Fe	Cu	Ni	Mg
2.7852	0.7925	0.5635	0.0032	0.0002	0.0003	0.0004	Balance

**Table 3:** Compressive characteristics of the magnesium alloy foam and magnesium composite foam.

	Yield Strength (MPa)	Plateau Stress (MPa)	Densification Strain	Energy Absorption (MJ/m^3^)	Ideal Energy Absorption Efficiency
Magnesium alloy foam	21.18	27.93	0.50	16.89	0.75
Magnesium composite foam	25.75	30.60	0.57	18.47	0.77

## References

[1] Xia X.C., Chen X., Zhang Z., Chen X.W., Zhao W.M., Liao B., Hur B.Y. (2014). Compressive properties of closed-cell aluminum foams with different contents of ceramic microspheres. Mater. Des..

[2] Orbulov I.N., Májlinger K. (2013). Description of the compressive response of metal matrix syntactic foams. Mater. Des..

[3] Altenaiji M., Guan Z.W., Cantwell W.J., Zhao Y., Schleyer G.K. (2014). Characterization of aluminum matrix syntactic foams under drop weight impact. Mater. Des..

[4] Májlinger K., Orbulov I.N. (2014). Characteristic compressive properties of hybrid metal matrix syntactic foams. Mater. Sci. Eng. A..

[5] Xia X.C., Zhang Z., Zhao W.M., Li C., Ding J., Liu C.X., Liu Y.C. (2017). Acoustic properties of closed-cell aluminum foams with different macrostructures. J. Mater. Sci. Tech..

[6] Banhart J. (2001). Manufacture, characterization and application of cellular metals and metal foams. Prog. Mater. Sci..

[7] Mondal D.P., Majumder J.D., Jha N., Badkul A., Das S., Patel A. (2012). Titanium-cenosphere syntactic foam made through powder metallurgy route. Mater. Des..

[8] Alizadeh M., Mirzaei-Aliabadi M. (2012). Compressive properties and energy absorption behavior of Al-Al_2_O_3_ composite foam synthesized by spaceholder technique. Mater. Des..

[9] Cox J., Luong D.D., Shunmugasamy V.C., Gupta N., Strbik O.M., Cho K. (2014). Dynamic and thermal properties of aluminum alloy A356/silicon carbide hollow particle syntactic foams. Metals.

[10] Rohatgi P.K., Kim J.K., Guo R.Q., Robertson D.P., Gajdardziska-josifovska M. (2002). Age-hardening characteristics of aluminum alloy-hollow fly ash composites. Metall. Mater. Trans. A.

[11] Balch D.K., O’Dwyer J.G., Davis G.R., Cady C.M., Gray G.T., Dunand D.C. (2005). Plasticity and damage in aluminum syntactic foams deformed under dynamic and quasistatic conditions. Mater. Sci. Eng. A.

[12] Tao X.F., Zhang L.P., Zhao Y.Y. (2009). Al matrix syntactic foam fabricated with bimodal ceramic microspheres. Mater. Des..

[13] Xia X.C., Feng J.L., Ding J., Song K.H., Chen X.W., Zhao W.M., Liao B., Hur B.Y. (2015). Fabrication and characterization of closed-cell magnesium-based composite foams. Mater. Des..

[14] Wen C.E., Yamada Y., Shimojima K., Chino Y., Hosokawa H., Mabuchi M. (2004). Compressibility of porous magnesium foam: dependency on porosity and pore size. Mater. Lett..

[15] Park S.H., Um Y.S., Kum C.H., Hur B.Y. (2005). Thermophysical properties of Al and Mg alloys for metal foam fabrication. Colloid. Surf. A.

[16] Xu Z.G., Fu J.W., Luo T.J., Yang Y.S. (2012). Effects of cell size on quasi-static compressive properties of Mg alloy foams. Mater. Des..

[17] Liu J.A., Yu S.R., Zhu X.Y., Wei M., Li S., Luo Y.R., Liu Y.H. (2008). The compressive properties of closed-cell Zn-22Al foams. Mater. Lett..

[18] Yang D.H., Hur B.Y., Yang S.R. (2008). Study on fabrication and foaming mechanism of Mg foam using CaCO_3_ as blowing agent. J. Alloy Compd..

[19] Lu G.Q., Hao H., Wang F.Y., Zhang X.G. (2013). Preparation of closed-cell Mg foams using SiO_2_-coated CaCO_3_ as blowing agent in atmosphere. Trans. Nonferrous Met. Soc. China.

[20] Mcrae J.D., Naguib H.E., Atalla N. (2010). Mechanical and acoustic performance of compression-molded open-cell polypropylene foams. J. Appl. Polym. Sci..

[21] Orbulov I.N. (2012). Compressive properties of aluminium matrix syntactic foams. Mater. Sci. Eng. A.

[22] Birla S., Mondal D.P., Das S., Kashyap D.K., Ch V.A.N. (2017). Effect of cenosphere content on the compressive deformation behaviour of aluminum-cenosphere hybrid foam. Mater. Sci. Eng. A.

[23] Myers K., Katona B., Cortes P., Orbulov I.N. (2015). Quasi-static and high strain rate response of aluminum matrix syntactic foams under compression. Compos. Part A Appl. Sci. Manuf..

[24] Maria J.A.S., Schultz B.F., Ferguson J.B., Gupta N., Rohatgi P.K. (2013). Effect of hollow sphere size and size distribution on the quasi-static and high strain rate compressive properties of Al-A380-Al_2_O_3_ syntactic foams. J. Mater. Sci..

[25] Daouda A., El-khair M.T.A., Abdel-Aziza M., Rohatgi P. (2007). Fabrication, microstructure and compressive behavior of ZC63 Mg-microballoon foam composites. Compos. Sci. Technol..

[26] Huang Z.Q., Yu S.R., Li M.Q. (2010). Microstructures and compressive properties of AZ91D/fly-ash cenospheres composites. Trans. Nonferrous Met. Soc. China.

[27] Kainer K.U. (2003). Magnesium Eigenschaften, Anwendungen, Potenziale.

[28] Yu M., Zhu P., Ma Y.Q. (2012). Experimental study and numerical prediction of tensile strength properties and failure modes of hollow spheres filled syntactic foams. Comp. Mater. Sci..

[29] Xia X.C., Zhao W.M., Wei Z.H., Wei Z.G. (2012). Effects of specimen aspect ratio on the compressive properties of Mg alloy foam. Mater. Des..

[30] Wang J., Zhang Z., Jiang Q., Xia X.C., Qiu C.R., Ding J., Zhao W.M. (2017). A novel bubble nucleation particle for magnesium composite foam. Mater. Lett..

[31] Mondal D.P., Goel M.D., Das S. (2009). Compressive deformation and energy absorption characteristics of closed cell aluminum-fly ash particle composite foam. Mater. Sci. Eng..

[32] Mondal D.P., Goel M.D., Das S. (2009). Effect of strain rate and relative density on compressive deformation behaviour of closed cell aluminum-fly ash composite foam. Mater. Des..

[33] Goel M.D., Peroni M., Solomos G., Mondal D.P., Matsagar V.A., Gupta A.K., Larcher M., Marburg S. (2012). Dynamic compression behavior of cenosphere aluminum alloy syntactic foam. Mater. Des..

[34] Goel M.D., Matsagar V.A., Gupta A.K., Marburg S. (2012). Strain rate sensitivity of closed cell aluminium fly ash foam. Trans. Nonferrous Met. Soc. China.

[35] Avalle M., Belingardi G., Montanini R. (2001). Characterization of polymeric structural foams under compressive impact loading by means of energy-absorption diagram. Int. J. Impact Eng..

[36] Bratt P.W., Cunnion J., Spivack B.D. (1983). Mechanical testing of glass hollow microspheres. Adv. Mater. Charact..

[37] Carlisle K.B., Lewis M., Chawla K.K., Koopman M., Gladysz G.M. (2007). Finite element modeling of the uniaxial compression behavior of carbon microballoons. Acta Mater..

[38] Li P., Petrinic N., Siviour C.R. (2012). Finite element modelling of the mechanism of deformation and failure in metallic thin-walled hollow spheres under dynamic compression. Mech. Mater..

[39] Wang H.F., Kong W.B., Hu P.X., Hu H. (2016). Effect of rare earth elements on microstructure and properties of as-cast AlMg_5_Si_1_ alloy. Foundry.

[40] Li J.C., Hao X.M., Wei A.L., Zhao H.F. (2008). Effect of Mg on Microstructure and Mechanical Properties of Zn-based Alloy. Hot Work. Technol..

[41] Emamy M., Jafari Nodooshan H.R., Malekan A. (2011). The microstructure, hardness and tensile properties of Al-15%Mg2Si in situ composite with yttrium addition. Mater. Des..

[42] Moussa M.E., Waly M.A., El-Sheikh A.M. (2014). Effect of Ca addition on modification of primary Mg_2_Si, hardness and wear behavior in Mg_2_Si hypereutectic alloys. J. Magnes. Alloy.

[43] Li C.F., Wei X.L., Zheng L., Yu W.B., Huang R.Q. (2016). Modifcation effect of calcium-magnesia phosphate fertilizer on microstructure and mechanical properties of Mg_2_Si/Mg-4Si composite. China Found..

[44] Rzychoń T., Janik R. (2015). The Influence of Calcium on the Primary Mg_2_Si Phase in the Hypereutectic Mg-Si Alloys. Solid State Phenom..

[45] Kim J.J., Kim D.H., Shin K.S., Kim N.J. (1999). Modification of Mg_2_Si morphology in squeeze cast Mg-Al-Zn-Si alloys by Ca or P addition. Scr. Mater..

